# Improving Mental Health Knowledge Among Brazilian Community Health Agents in a Training Program for a Self‐Reported Mental Health Assessment

**DOI:** 10.1111/phn.70061

**Published:** 2025-12-26

**Authors:** Matheus R. Dornelles, Sheila G. Câmara, John P. Hirdes, Jamila Geri Tomaschewski Barlem, Thomas Heimann, Alice Hirdes

**Affiliations:** ^1^ Unidade de Pronto Atendimento (UPA) from Gravataí Santa Casa de Porto Alegre Porto Alegre Rio Grande do Sul Brazil; ^2^ Graduate Program in Psychology and Health Federal University of Health Sciences of Porto Alegre Porto Alegre Rio Grande do Sul Brazil; ^3^ School of Public Health Sciences University of Waterloo Waterloo Canada; ^4^ Chair of the interRAI's International Network of Excellence in Mental Health and the interRAI Network of Canada Fellow of the Canadian Academy of Health Sciences and the Royal Society of Canada Ontario Canada; ^5^ Graduate Program in Nursing Federal University of Rio Grande Rio Grande Rio Grande do Sul Brazil; ^6^ Universidade Luterana do Brasil Canoas Rio Grande do Sul Brazil; ^7^ Graduate Program in Nursing Federal University of Rio Grande Rio Grande Rio Grande do Sul Brazil; ^8^ Full Fellow of the interRAI Network for Mental Health and Member of the interRAI Instrument and System Development Committee Porto Alegre Rio Grande do Sul Brazil

**Keywords:** attitudes, Community Health Agents, feasibility studies, health knowledge, interRAI, mental health, practice, Primary Health Care

## Abstract

**Objectives:**

This study aimed to evaluate a mental health training process that included the implementation of the *interRAI Self‐Reported Assessment for Mental Health* (*SAMH*) tool among Community Health Agents (CHAs), to improve knowledge of mental health issues, as well as to assess the tool's feasibility and acceptability.

**Method:**

This is a quasi‐experimental study. The participants were 24 CHAs from a municipality in the Metropolitan Region of Porto Alegre, Rio Grande do Sul, Brazil. The instruments used were a sociodemographic and work‐related questionnaire; a pre‐test and post‐test questionnaire to assess the knowledge of mental health, and a questionnaire assessing the feasibility and accessibility of interRAI SAMH, which was part of the training process. To compare knowledge before and after the process, a paired‐sample *t*‐test was used. Feasibility and acceptability of the SAMH were evaluated using a questionnaire and qualitative data.

**Results:**

The training, combined with the SAMH data collection process, contributed to increased perceived knowledge of the following topics: psychiatric reform, human rights in mental health, Mental Health National Policy, Mental Health Care Models, Psychiatric Reform Law, risk factors for suicide, depression, alcohol and drugs, personalized therapeutic plan, psychoses, anxiety disorders, psychiatric urgences and emergencies, and interdisciplinary work. The results of feasibility and acceptability evidenced that CHAs feel more capable of recognizing psychiatric pathologies and emergencies, such as individuals at risk of suicide.

**Conclusion:**

The findings support the feasibility and acceptability of the interRAI SAMH tool and its potential to enhance CHAs’ knowledge and ability to identify and refer individuals with mental health conditions in Primary Health Care.

## 1 Introduction

Primary Health Care (PHC), the entry point for users into Brazil's Unified Health System (SUS), is responsible for developing prevention and health promotion actions for the population at the individual and collective levels (Brasil 2017). For this, Community Health Agents (CHAs) are a workforce in PHC and the Family Health Strategy (ESF) (Jatobá et al. 2020). The CHA model used in Brazil is a promising example for international application, as these professionals' work has shown clear evidence of a positive impact on the health of the population served (Exemplars in Global Health 2025; Giugliani et al. 2021). CHAs must be directly integrated into their communities, residing in the area where they work, standing out for their communication and interpersonal relationships, and serving as community leaders (Jatobá et al. 2020).

These professionals are particularly prominent in low and middle‐income countries, especially in Africa, Asia, and Latin America, with few experiences in high‐income countries in North America and Oceania (de D Méllo et al. 2023). Internationally, the Exemplars in Global Health project identified four countries, Bangladesh, Ethiopia, Liberia, and Brazil, as successful experiences with CHAs (Exemplars in Global Health 2025). In Brazil, it is estimated that there are more than 400,000 CHAs working across the country, according to data from the Brazilian government (Brasil 2023).

In Brazil, CHAs represent an important workforce for the early identification and management of individuals with mental disorders in PHC. Law No. 13.595 of 2018 includes important duties for these professionals, among them, performing regular and periodic visits to monitor cases of psychological distress or dependency on tobacco, alcohol, or other drugs (Brasil 2018). In PHC, the CHAs are essential to ESF teams. Law 11.350 of 2006, amended in 2018 by Law No. 13.595, establishes important milestones for these professionals' work within SUS, making them mandatory members of the teams. CHAs have the crucial role of preventing and promoting health, working directly in the territories of assigned areas, promoting individual and collective actions through popular health education references (Brasil 2018).

The introduction of these professionals into PHC raises an important concern regarding service users, with a focus on achieving equity in access to health care for the most vulnerable populations (Nepomuceno et al. 2021). Efforts to achieve effective strategies for mental health, with care practices modified by the advent of Brazilian Psychiatric Reform, highlight the need for comprehensive individual care at various services at the Psychosocial Care Networking (RAPS), such as admissions in crisis at general hospitals, Psychosocial Rehabilitation Centers (CAPS) and PHC. For this, is essential to develop a clinical practice grounded in multidisciplinary collaboration and in the provision of care tailored to the specific needs of service users, providing quality care with an emphasis on continuous care and follow‐up (Coutinho et al. 2024). In this sense, CHAs function as a connecting link between service users and health services, actively conveying users’ needs to the health care teams (Cardoso et al. 2020). The stability of PHC teams and the relationship with users are predominant factors in the early identification of mental disorders (Salgado and Fortes, 2021).

Despite the relevance of CHAs' work, studies (Lima et al. 2022; Marinho and Bispo Júnior, 2020) highlight the need for initiatives to improve working conditions and reduce workload, insufficient institutional support, and targeted training processes for controlling diseases. Insufficient professional training has been reported as a factor contributing to health agents' stress and insecurity (de HC Barreto et al. 2018). Specifically in mental health, research (Salgado et al. 2023; da Silva Júnior et al. 2021) has highlighted the need for professional training as a challenge to be overcome to strengthen mental health care. Therefore, continuing education opportunities are essential for effective collaboration of these professionals in population health care in various care lines, providing more expertise and a clearer definition of their scope of work (Petruzzi et al. 2024). However, despite the relevance of CHAs’ work, evidence shows that training opportunities for these professionals are scarce, particularly in the field of mental health.

Given the need for mental disorder screening using an instrument appropriate for lay assessors, the interRAI international research consortium developed the interRAI Self‐Reported Assessment for Mental Health (SAMH). The SAMH has 100 clinical items that address mental health issues, psychosocial functioning, physical health, economic and environmental factors, informal supports, quality of life, and perceived needs. This tool builds on interRAI's experience with other clinician‐administered and self‐reported assessment systems for mental health and PHC (Iheme et al. 2022; Luo et al. 2021).

The SAMH is being used in pilot studies in several countries across Latin America, Central America, the Caribbean, Europe, Oceania, Asia, and North America to explore its feasibility, acceptability, and cultural appropriateness among people with mental health needs. Although it can be used by health services employing lay mental health assessors, its use is not limited to them. In Brazil, the tool was administered by CHAs and is currently undergoing validation.

### 1.1 General Objective

This study aimed to evaluate a mental health training process that included the implementation of the *interRAI Self‐Reported Assessment for Mental Health* (*SAMH*) tool to CHAs to improve knowledge of mental health issues, as well as to assess the tool's feasibility and acceptability.

## 2 Method

### 2.1 Design, Population, Sample, and Period

The study has a quasi‐experimental design and an intentional sample. The target population consisted of CHAs linked to a municipality in the Metropolitan Region of Porto Alegre, Brazil, working in the FHS. The municipality has 28 PHC units, with a total of 301 CHAs working in the teams. Considering the FHS model, four units that did not fit the proposed methodology were excluded because they did not have teams of this model. After a meeting with municipal health managers to implement the study, one CHA per health unit was designated to participate in our study, the PHC unit could not spare more than one CHA because of other work they were doing in other public health responses. Thus, one CHA was selected per unit, by unit managers for their identification with the mental health field, resulting in a sample of 24 CHAs.

A pre‐test was conducted in October 2023 before using SAMH. Afterwards, a capacity‐building session on mental health topics was conducted, followed by data collection using the SAMH. At the end of the data collection phase, a post‐test was applied. After completing all these steps, the SAMH evaluation instrument was administered in June 2024 to assess its feasibility and acceptability.

### 2.2 Training in Mental Health Knowledge

The initially planned training of 50 h was reduced at the management's request. Thus, the scheduled content was reduced to 8 h prioritizing topics that emerged from needs expressed by CHAs: Psychiatric Reform in Brazil, mood disorders; anxiety disorders, psychiatric emergencies, with a focus on suicide prevention.

It took place in person, at a location previously defined by the foundation that hires the CHAs in October 2023. The training's objective was explained: knowledge and procedures for applying SAMH. At this time, all items of the tool were read and discussed. The data collection process was weekly monitored by the field coordinator during the visits to the units. During the supervisions, questions and doubts were clarified, empirically complementing the training.

The initially planned 50‐h training was reduced at the request of the management and redesigned to an 8‐h format, according to the possibilities offered. It was conducted in person at a location previously defined by the foundation responsible for hiring the CHAs, in October 2023. The first part addressed mental health topics that emerged from needs expressed by the CHAs, including the Brazilian Psychiatric Reform, mood disorders, anxiety disorders, and psychiatric emergencies, with an emphasis on suicide prevention. The second part focused on the knowledge and procedures required for administering the interRAI SAMH tool. During this stage, all items of the instrument were read and discussed in detail.

The data collection process was systematically monitored by the field coordinator. During the supervision meetings, questions and doubts were clarified, empirically complementing the training.

### 2.3 Instruments

The instruments used were: (a) a form with sociodemographic and work‐related data of CHAs; (b) a pre‐test and post‐test questionnaire to assess perceived knowledge in mental health before and after training; and (c) an instrument for evaluating the feasibility and acceptability of SAMH.

### 2.4 Form With Sociodemographic and Work‐Related Data

The instrument consisted of eight quick‐fill questions, which could be answered online, with an estimated time of 2 min. The questions covered sociodemographic aspects such as gender, age, and educational level. Regarding work‐related aspects, the questions addressed the professional's experience as CHAs; previous mental health training with a minimum workload of 40 h; daily work involving patients with mental disorders or emotional distress; and the need for mental health training with a minimum workload of 40 h.

### 2.5 Mental Health Perceived Knowledge Assessment Questionnaire

After selecting CHAs, a pre‐test with questions related to mental health was applied, with content covering 20 items on psychiatric reform, pathologies frequently encountered in PHC patients, such as mood disorders, anxiety disorders, psychoses, and psychiatric emergencies, with a focus on identifying and managing suicide risk. The test was created by three professionals: a nurse from an Emergency Care Unit (UPA), a nurse with expertise in mental health, and a community psychologist. Some examples about the questions: What do you know about the human rights of people with mental disorders? What do you know about Bipolar Disorder? What is your knowledge about the risk factors for suicide? What is your knowledge about alcohol and other drug use? The response options were: 0 (not at all), 1 (a little), 2 (quite a bit), and 3 (very much). In this study, reliability coefficients were calculated using Cronbach's alpha and McDonald's omega for the questionnaire. Values of *α* = 0.96 and *ω* = 0.96 were obtained.

### 2.6 interRAI Self‐Reported Assessment for Mental Health (SAMH)—Feasibility and Acceptability Study

The evaluation instrument “interRAI Self‐Reported Assessment for Mental Health (SAMH)—Feasibility and Acceptability study”, was created by the international coordinator of the pilot research and translated into Portuguese. This feasibility and acceptability evaluation (Damian et al. 2020; Iheme et al. 2022) will serve as a basis for refining SAMH for future use in mental health services in Brazil and other Latin American countries.

This instrument consists of eight questions: (1) How long did it take you to complete the interRAI SAMH? (2) How difficult was it for you to complete the interRAI SAMH? (3) Which questions were the most difficult for you to complete? (4) Did you find any questions embarrassing or offensive? (5)Which questions did you find embarrassing or offensive? (6) How well did the interRAI SAMH identify your current needs? (7) What information was missing from the interRAI SAMH that is important for your current needs? 8. Which questions would you delete from the interRAI SAMH?

### 2.7 Data Collection

Prior to the training, an initial assessment of the topics of the training was carried out with the CHAs, to identify the topics they need more knowledge, considering the reduction of the time. During the training, initially, a form with sociodemographic and work‐related data of CHAs and the mental health perceived knowledge assessment questionnaire—pre‐test—was applied. This phase was followed by training and clarification sessions addressing the main mental disorders and suicide risk management. Only after these sessions, at the end, the training on the completion of the SAMH instrument was conducted. It was applied by the 24 CHAs between October 2023 and April 2024 to 524 participants, with an average completion time of 35 min. After training and SAMH data collection in the field, the mental health knowledge assessment questionnaire—post‐test—was reapplied. At this time, the feasibility and acceptability (Abildgaard et al. 2016) evaluation instrument of SAMH was also applied. Finally, a group meeting was held for a general evaluation of the process.

The qualitative data were obtained through monitoring the SAMH data collection process in the field (process evaluation) (Moore et al. 2015) and the final evaluation. The process evaluation aimed to monitor the dynamics of procedures to improve their quality, identify possible unexpected or secondary effects, and contribute to interpreting the quantitative data (Tejada et al. 2014). It was conducted by the field coordinator researcher through weekly meetings with CHAs. The information obtained was recorded in a field diary (Flemming et al. 2019). The final qualitative evaluation corresponded to the last group meeting, where the experience in the process and its overall evaluation in terms of mental health knowledge were addressed (McGill et al. 2020). All data, quantitative and qualitative, were deidentified.

### 2.8 Data Analysis

Quantitative and qualitative data were used to explore CHAs' knowledge in mental health before, during, and after the training process and SAMH application experience (Abildgaard et al. 2016). Quantitative data, referring to participants' characterization, the Mental Health Perceived Knowledge Assessment Questionnaire (pre‐ and post‐tests), and the SAMH Feasibility and Acceptability Questionnaire were entered and analyzed in JASP 0.19.3.

Initially, a descriptive analysis was conducted. A paired‐samples *t*‐test was used, and the Wilcoxon signed‐rank test was applied for non‐parametric data. Effect size was assessed using the rank‐biserial correlation, which follows Cohen's guidelines for correlation coefficients. The significance level considered was 0.05.

To analyze the qualitative data, it was used a categorical analysis. The data were organized into categories based on the items from the perceived knowledge test: mental health in Brazil; psychiatric urgencies and emergencies; mental health disorders; PHC; addictions; human rights; mental health care models; violence in mental health; individualized therapeutic project; and interdisciplinary work. Those categories were supplemented by field findings from the data collection. This qualitative information provided a deeper context for understanding the quantitatively assessed training process.

### 2.9 Ethical Aspects

This study was approved by the Ethics Committee of the Lutheran University of Brazil (no. 6, 278,523), according to Resolution n. 466/12. All patients signed the Informed Consent Form.

## 3 Results

Table 1 shows sociodemographic and professional characteristics of participants.

**TABLE 1:** Sociodemographic and professional characteristics of participants (*N* = 24).

Variable	F	%
Age (years)		
34–45	10	41.7
46–55	6	25.0
56–71	8	33.3
Gender		
Female	21	87.5
Male	3	12.5
Time of experience as community health agent (years)		
1–4	2	8.3
5–9	12	50.0
10–15	10	41.7
Previous mental health training (≥40 h)		
Yes	5	20.8
No	19	79.2
Provides care to people with mental disorders or psychological distress		
Yes	22	91.7
No	2	8.3
Perceived need for extended mental health training (≥40 h)		
Yes	21	87.5
No	3	12.5
Educational level		
Completed high school	17	70.8
Undergraduate in progress	3	12.5
Completed undergraduate degree	4	16.7

Concerning the evaluation of the training effect, Table 2 presents the items addressed in the pre‐ and post‐tests. Descriptive information of the items (means—M and standard deviations—SD) is also presented.

**TABLE 2:** Descriptive measures and comparison of means of CHA perceived knowledge on mental health items pre‐ and post‐training and SAMH application (*n* = 24). Canoas, RS, 2024.

	POST		PRE				
	M	SD	M	SD	W	*z*	*p*
1. Psychiatric Reform	1.04	0.55	0.58	0.58	104.00	2.49	0.002
2. Human Rights	1.33	0.56	0.92	0.58	45.00	2.66	0.002
3. Mental Health National Policy	1.17	0.56	0.63	0.65	78.00	3.05	<0.001
4. Mental Health Care Models	1.21	0.51	0.88	0.61	28.00	2.36	0.007
5. Law n° 10.216/2001	1.08	0.72	0.42	0.58	113.50	3.03	<0.001
6. Risk factors to suicide	1.58	0.72	1.33	0.82	36.00	1.59	0.048
7. Bipolar Disorder	1.50	0.66	1.33	0.56	27.00	1.26	0.091
8. Primary Health Care	1.54	0.66	1.38	0.71	44.00	0.97	0.154
9. Depression	1.63	0.65	1.38	0.65	36.00	1.59	0.048
10. Psychosocial Care Network	0.92	0.83	0.79	0.78	42.00	0.80	0.196
11. Psychosocial Care Network—Canoas	1.08	0.83	0.88	0.80	54.00	1.17	0.106
12. Matrix Support	1.33	0.70	1.21	0.66	30.00	0.88	0.175
13. Harm Reduction Policy	1.13	0.95	1.04	0.81	45.50	0.51	0.297
14. Alcohol and drugs	1.71	0.69	1.46	0.59	44.00	1.68	0.033
15. Violence and Mental Health	1.42	0.72	1.29	0.75	44.00	0.97	0.154
16. Personalized Therapeutic Plan	1.04	1.00	0.58	0.83	99.00	2.21	0.009
17. Psychoses	1.08	0.58	0.75	0.85	40.50	2.13	0.012
18. Anxiety Disorders	1.42	0.72	1.13	0.61	21.00	2.20	0.013
19. Psychiatric Urgencies and Emergencies	1.33	0.64	0.83	0.92	97.50	2.82	<0.001
20. Interdisciplinary Work	1.38	0.92	0.88	0.99	55.00	2.80	0.002
TOTAL QUESTIONNAIRE	1.29	0.54	0.98	0.51	232.00	3.42	<0.001

It was found that the participants' mean responses to the 20 items improved in the post‐test, indicating an increase in perceived knowledge. There were 13 items where there was a significant difference between the pre‐ and post‐test means. In this sense, the training, together with the data collection process with the SAMH, significantly contributed to greater knowledge. Regarding the overall average of the 20 items on the post‐ and pre‐test questionnaire, the difference was also significant, indicating greater overall knowledge of the topics addressed. In terms of the general indices of the pre‐ and post‐test evaluation questionnaire, the effect size was calculated using rank‐biserial correlation, which was 0.83, considered high.

According to the range of response options to the instrument (0: not all; 1: a little; 2: quite a bit; and 3: very much), it was found that the participants' mean responses, both in terms of items and the overall instrument, in the post‐test, the significant scores in most items remained around “a little”, with a few items having a mean close to “quite a bit”. In the pre‐test were around “a little”.

### 3.1 Qualitative Feedback From the interRAI Self‐Reported Assessment for Mental Health

In the qualitative evaluation of SAMH use, the CHAs reported that the systematic use of this tool enabled learning and the formulation of specific questions about mental health issues and mental disorders that were previously not asked. They also reported feeling more capable of recognizing psychiatric pathologies and emergencies, such as individuals at risk of suicide.

CHA1 reported that the interviews helped identify emotional vulnerabilities in individuals and their families. They also mentioned that the formulation of the questions allows for responses that denote the severity of the suffering everyone is experiencing. This, in turn, enables these cases to be brought to the attention of the multidisciplinary team at the unit, allowing for quicker case management.

CHA2 said, “I was surprised that the people around me, who were always smiling, were actually in psychological distress.” CHA3 stated that after applying SAMH, they became more attentive to the day‐to‐day details of PHC users with mental disorders or mental distress. They mentioned that these individuals often feel like a burden to their families, undergoing a process of isolation, even giving up on medication treatments or seeking help. They reported statements from patients like, “nothing is worth it anymore,” with a worsening of the condition. After systematically applying SAMH, CHA3 reported feeling unprepared they had been, as a professional, to handle mental health cases, stating that investment in training is essential for improving care for these patients.

CHA4 said they had attended users with self‐harm intentions and suicidal ideation, and after applying SAMH, they understood the need to address the issue with the patient and began asking the questions included in the tool. CHA5 said, “Most of the achievements in medicine were and are based on research, evaluating behaviors, ideas, mental, emotional health, and many others… The development of this research made us reflect both in our professional and personal lives. In the professional realm, it brought warning signs, making it very clear the need for specific training in mental health, so that we, frontline professionals, can work more calmly, as we are inside homes daily and see how much the mental health of family members impacts and often disrupts the family.”

CHA6 reported that “At first, the approach was difficult. But as time went on, the users in the area I serve began to understand and accept the importance of the research… That's why I found the research experience important and very meaningful, so I can deal with these situations in my daily work.”

CHA7 said, “I noticed that the interviewed individuals initially resist, but after a while, they start to feel secure and begin to open up a bit more. There are cases where they feel welcomed to the point of discussing issues that go beyond the interview…”

### 3.2 Feasibility and Acceptability of the interRAI Self‐Reported Assessment for Mental Health

The results of the SAMH evaluation regarding the concepts of acceptability and feasibility, based on the instrument developed by the coordinator of the international pilot project and translated into Portuguese, showed that most CHAs took between 30–44 min (*n* = 14) and 45–59 min (*n* = 7) to complete the assessment. Only three CHAs required 1 h or more. Regarding the level of difficulty, eight CHAs reported that completing the instrument was not difficult, six reported that it was slightly difficult, and ten considered it moderately difficult. Among all participants, fifteen reported having no difficulty with any specific item, while nine reported difficulties.

In relation to whether information was missing from the tool, 18 CHAs reported that no information was missing, and regarding suggestions, six CHAs offered the following: that SAMH could be more objective, include guidance on where to seek help, have more items on the last few days, include questions about the social environment or who the patient interacts with, be more specific about mental illnesses, and provide information about services available in the municipal network.

Regarding the question “Which questions would you exclude from the interRAI SAMH?”, 19 CHAs said they would not exclude any, two CHAs would exclude questions with the same content and purpose, one CHA would exclude questions about the use of psychoactive substances, and one CHA would exclude the section on suicide because it could trigger negative emotions.

## 4 Discussion

This study aimed to evaluate a mental health training process that included the implementation of the interRAI SAMH tool to CHAs, to improve the knowledge of mental health issues, as well as to assess the tool's feasibility and acceptability. Our quantitative results evidence an increase in perceived knowledge in terms of the following topics: psychiatric reform, human rights in mental health, Mental Health National Policy, Mental Health Care Models, Law N^°^ 10.216/2001 (Psychiatric Reform Law), risk factors for suicide, depression, alcohol and drugs, personalized therapeutic plan, psychoses, anxiety disorders, psychiatric urgences and emergencies, and interdisciplinary work.

In this sense, consistent with the results of national research (Amaral et al. 2018; Cordeiro et al. 2020; de FO Faria et al. 2020; Moro et al. 2020; de HC Barreto et al. 2018,) and international studies (Asher et al. 2021; Naslund et al. 2019; Petruzzi et al. 2024; Singla et al. 2019; Tyagi et al. 2023), training aids in the practice of CHAs and lay assessors in attending to users with mental disorders or emotional distress. Likewise, the use of instruments and protocols applied systematically promotes learning (Asher et al. 2021), a situation that occurred in this research with the repeated application of SAMH by CHAs. Findings from the field diary and final evaluation indicated that CHAs enhanced their ability to recognize individuals experiencing emotional distress, mental disorders, or risk situations, reinforcing the importance of using a screening instrument and the need for ongoing training in this field. The work in multiprofessional teams, including the work of CHAs alongside professionals through interdisciplinary actions, benefits the user with mental disorders in the PHC network and promotes social participation and the individual's involvement in their care process. The multidisciplinary health team model brings more cohesion to the treatment process, involving professionals with multiple perspectives (Moro et al. 2020).

The results of our study demonstrated a meaningful increase in CHAs’ perceived knowledge, particularly regarding how to approach individuals at risk and how to demystify myths surrounding suicide. However, the suggestion to exclude suicide‐related items from the training instrument (SAMH), based on the assumption that such questions could act as a trigger, is misguided. This view reflects broader difficulties in addressing suicide, limited knowledge about myths and facts related to the topic, and, ultimately, the need for targeted training in this area. In Brazil, suicide prevention has been formally addressed through public policy: since 2014, a ministerial ordinance has required municipal health authorities to report suicide attempts within 24 h, and in 2019, a law established the National Policy for the Prevention of Self‐Mutilation and Suicide (World Health Organization 2021). In this context, CHAs are required to report cases to PHC teams, underscoring the need for adequate knowledge and training on this issue.

The findings from the pre‐test and the feasibility and acceptability assessment are aligned with results and with the issues raised during on‐site supervision. Our results showed the need to increase the knowledge about the components of the Psychosocial Care Network in Brazil, and specifically in the municipality's Psychosocial Care Network. The knowledge of the different services is important to refer patients in crises to the secondary or tertiary level, Psychosocial Care Centers or general hospitals, respectively. It should be emphasized that developing guides to support knowledge of the Psychosocial Care Network, available services, and referral pathways lies within the scope of PHC professionals’ responsibilities. A study found that the frequency of mental health care in PHC, as well as referrals to specialized services, was low (Alcântara et al. 2020).

The results highlighted the need for knowledge acquisition and the active participation of CHAs in mental health matrix support within PHC. Engagement in clinical case discussions with PHC teams and mental health support consultants with greater technical expertise may further facilitate this process. As reported in previous research (Amaral et al. 2018), mental health matrix support from the perspective of CHAs has proven to be an effective strategy, promoting changes in professional attitudes, increasing access to services, fostering the development of new care practices, and enhancing problem‐solving capacity. However, other research (de FO Faria et al. 2020) has shown that, despite the implementation of matrix support for PHC professionals in addressing alcohol and other drug use, fear, moral concerns, and stigma persist. Furthermore, these findings underscore the need for greater inclusion of adequately trained CHAs in matrix support activities, supported by continuing education to empower them to provide care to individuals with mental disorders (Alcântara et al. 2020).

Our results show that, despite their years of experience and the fact that most CHAs provide care to people with mental health issues, the majority have never received substantial training in mental health, although most perceive such training as necessary. An analysis of the scope of CHAs’ work in Ceará State—where the first experience of institutionalizing the Community Health Agents Program (PACS) took place—identified deficiencies in technical training, limited work support, and exposure to violence as factors constraining professional practice. Continuing education and participatory management were identified as potential enabling factors (de HC Barreto et al. 2018).

Data collection using the SAMH was preceded by face‐to‐face training on the use of the tool, in addition to weekly supervision of CHAs at the Health Units, which contributed to their performance. Another study (Brasil 2018) examining the effectiveness of workshops in increasing CHAs’ knowledge of the needs of users with mental disorders or psychological distress demonstrated improved understanding of the Psychosocial Care Network (RAPS) and current treatment processes. In contrast, a Popular Health Agents course comprising a total of 80 classroom hours found that administering questionnaires was challenging. The authors concluded that the questionnaire should have been studied in class after selecting a subgroup of participants to act as field researchers, providing them with specific training and remuneration for this task (Niemeyer et al. 2024).

Our study showed, through the feasibility and acceptability assessment, that some topics are perceived by CHAs as sensitive or embarrassing, such as alcohol and other drug use, which is consistent with previous research (de FO Faria et al. 2020). The suggestion to exclude questions related to psychoactive substance use is not appropriate, as substance use represents a concrete reality and a relevant issue in many areas of the municipality. These findings indicate that additional preparatory training focused on effective and sensitive approaches to discussing substance use may be warranted.

A research (Salgado and Fortes, 2021) aimed at assessing the detection of mental disorders in PHC identified important weaknesses, particularly in the identification of harmful alcohol use and Bipolar Mood Disorder. Another research (Alcântara et al. 2020) examining the identification of individuals with mental disorders and substance use by CHAs found that reports of mental disorder cases increased sixfold, even in the absence of training, following the closure of a psychiatric hospital. However, the identification of psychoactive substance users remained low, resulting in underreporting.

The feasibility and acceptability findings also demonstrated that CHAs were able to recognize individuals experiencing mental health distress after using the SAMH to interview participants. Similarly, research (Medeiros et al. 2020) examining CHAs’ attitudes before and after mental health training showed that training led to more positive attitudes regarding their ability to manage patients with mental health needs. Due to their work within the community and their close ties with families, CHAs are well‐positioned to identify and refer individuals to specialized services and to notify suicide attempts. Nevertheless, the evidence highlights the ongoing need to address myths and facts related to suicide. Research (de Almeida et al. 2021) identified beliefs about suicidal behavior that may negatively influence care for individuals at risk, reinforcing the importance of continued education and training in suicide prevention within PHC.

Reports from CHAs revealed fear of asking about drug use in neighborhoods controlled by drug trafficking, possibly because many CHAs live in the same territory and because the majority are women. Conversely, they were able to bring identified situations—such as suicide risk, violence, and relapse of mental disorders—into team discussions, which facilitated the identification of individuals requiring care and strengthened their sense of professional support. Working in the community with individuals with mental disorders has also been associated with feelings of threat and insecurity during home visits, as well as significant challenges in establishing boundaries between work and personal life due to the intensity of CHAs’ work demands (Leme et al. 2023). Although contextual factors related to the territory play an important role, CHAs’ insecurity is not solely attributable to territorial characteristics but also to the lack of collective work processes, which can lead to emotional strain and vulnerability among agents (Almeida et al. 2019).

Despite the international recognition of the role of CHAs in Brazil, this professional category remains undervalued at the national level. Insecurity, occupational risks, exposure to violence, inadequate working conditions, low wages, and insufficient support in terms of training and supervision have been identified as key weaknesses (Almeida et al. 2019; de HC Barreto et al. 2018; Vieira‐Meyer et al. 2022). These challenges were also reported by CHAs participating in this study during the SAMH data collection process. In Brazil, nurses are primarily responsible for the training and supervision of CHAs. Although the ESF within PHC is grounded in a comprehensive model of care, most health professionals remain insufficiently prepared to provide care for people with severe mental disorders living in the community (Alcântara et al. 2020; de FO Faria et al. 2020; Salgado et al. 2023; da Silva Júnior et al. 2021). Consistent with these findings, a national survey of registered nurses in the United States reported mental health training as the most frequently identified priority among PHC nurses (Castner et al. 2023).

### 4.1 Study Limitations

One limitation of this study concerns the initial training, which was originally planned for 50 h but had to be reduced at the request of local management. In addition, the quasi‐experimental design, combined with a relatively small sample size and the absence of a control group, limits the ability to attribute observed changes in perceived knowledge exclusively to the intervention, as other contextual factors may have influenced the outcomes.

### 4.2 Final Considerations

The training, combined with the systematic use of a mental health assessment tool and ongoing supervision, contributed to improving CHAs’ perceived knowledge in mental health. In their daily work, CHAs routinely deal with crises, psychological distress, and mental disorders, often with a limited theoretical background, which can result in underreporting of mental disorders and gaps in care. These findings highlight the importance of continuing education processes in supporting CHAs’ professional practice. In addition, training may help reduce stigma by increasing sensitivity toward the care of people with mental disorders and individuals who use psychoactive substances, thereby contributing to improved identification and management of these conditions within PHC.

The study also showed that the use of the SAMH facilitated the assessment of the severity and nature of psychiatric disorders, supported the development of targeted questions, enabled the identification of emotional vulnerabilities, and promoted the referral and discussion of cases within PHC teams. Given that the systematic use of the SAMH increased perceived knowledge, improved the recognition of individuals with symptoms and risk factors, and enhanced case management, the adoption of standardized mental health assessment protocols in PHC is recommended. In Brazil, public mental health services are organized through the Psychosocial Care Network (RAPS), which requires collaborative relationships across different levels and types of care. In this context, the effective inclusion of CHAs in mental health matrix support within PHC is also recommended, as this work methodology is particularly relevant for low‐ and middle‐income countries.

## Author Contributions

Matheus R. Dornelles contributed to the conceptualization, data curation, investigation, and writing. Sheila G. Câmara contributed to formal analysis, methodology, and writing. John P. Hirdes contributed to conceptualization, project administration, data curation, and review. Jamila Geri Tomaschewski Barlem writing, review, and editing. Thomas Heimann contributed to project administration and supervision, and review. Alice Hirdes contributed to project administration and supervision, writing, review, and editing. All authors reviewed the final version of the manuscript and approved the version for publication.

## Funding

This study was supported by interRAI.

## Ethics Statement

This study was approved by the Ethics Committee of the Lutheran University of Brazil (no. 6, 278,523), according to Brazilian Resolution n. 466/12.

## Consent

All patients signed the Informed Consent Form, according to the Brazilian Resolution no. 466/12.

## Conflicts of Interest

The authors declare no conflicts of interest.

## Data Availability

The data that support the findings of this study are available from the corresponding author upon reasonable request.
